# Supplementary material for: Ingenious Fabrication of Ag-Filled Porous Anodic Alumina Films as Powerful SERS Substrates for Efficient Detection of Biological and Organic Molecules
Source: Biosensors (Basel). 2022 Sep 29;12(10):807. doi: 10.3390/bios12100807 (PMC9599633; doi:10.3390/bios12100807)
Supplement: Supplementary file 1 [file biosensors-12-00807-s001.zip › biosensors-1884702-supplementary.pdf]

Supplementary Materials

Ingenious Fabrication of Ag-Filled Porous Anodic Alumina Films as Powerful SERS Substrates for Efficient Detection of Biological and Organic Molecules

Chih-Yi Liu [1], Rahul Ram [1,2], Rahim Bakash Kolaru [1,2], Anindya Sundar Jana [2], Annada Sankar Sadhu [1,2], Cheng-Shane Chu [1,3], Yi-Nan Lin [2], Bhola Nath Pal [4], Shih-Hsin Chang [5] and Sajal Biring [1,2,*]

[1] Organic Electronics Research Center, Ming Chi University of Technology, New Taipei City 24301, Taiwan
[2] Department of Electronic Engineering, Ming Chi University of Technology, New Taipei City 24301, Taiwan
[3] Department of Mechanical Engineering, Ming Chi University of Technology, New Taipei City 24301, Taiwan
[4] School of Material Science and Technology, Indian Institute of Technology, BHU, Varanasi 221005, India
[5] MSSCORPS Co., Ltd., Hsinchu 300047, Taiwan
* Correspondence: biring@mail.mcut.edu.tw

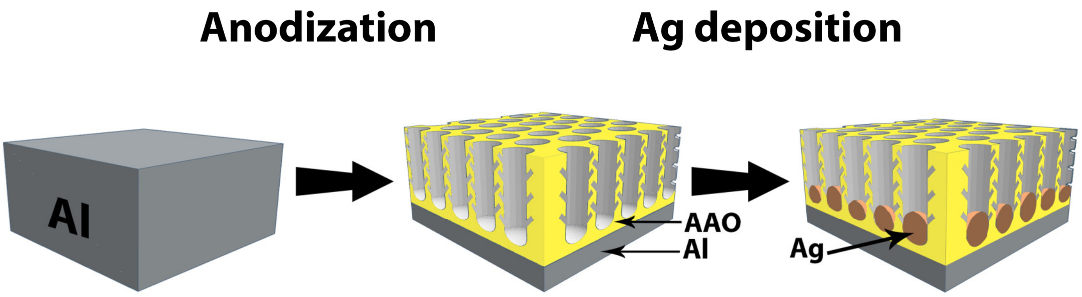

Figure S1. Schematic diagram showing the fabrication of Ag-AAO SERS substrates.

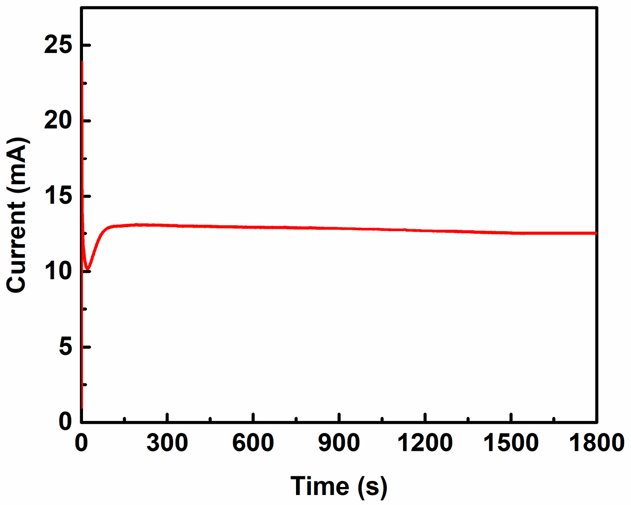

Figure S2. Current vs time curve in a typical anodization process.

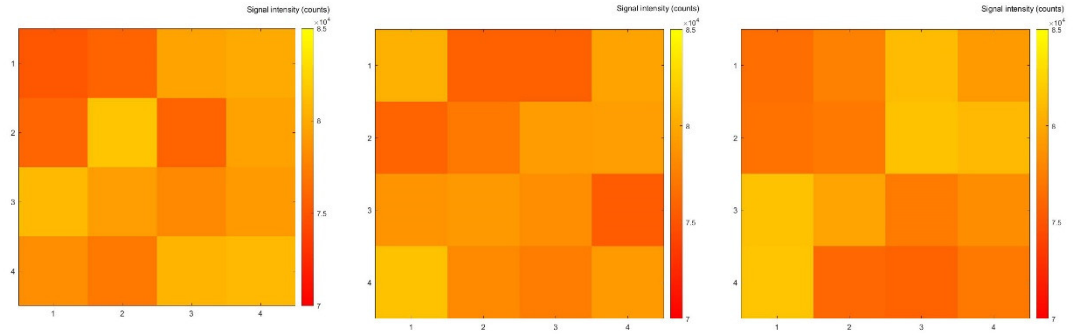

Figure S3. Three arbitrarily selected Raman mappings of 10^{-5} M R6G on an Ag-AAO substrate. Each mapping is performed by measuring spectra from a 4×4 square lattice with a lattice constant of 40 μm. The peak intensity of 613 cm^{-1} is selected for the mappings.

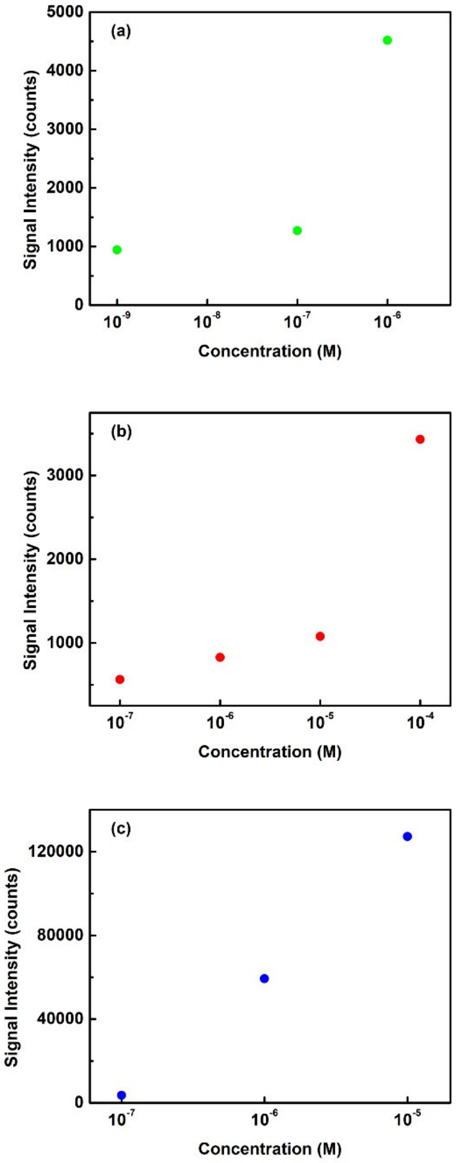

Figure S4. The plots of SERS signal intensities as functions of analyte concentrations based on the spectra in Figure 7. The analytes are (**a**) eosin Y (peak at 1622 cm^{-1}), (**b**) adenine (peak at 734 cm^{-1}), and (**c**) methylene blue (peak at 1625 cm^{-1}).

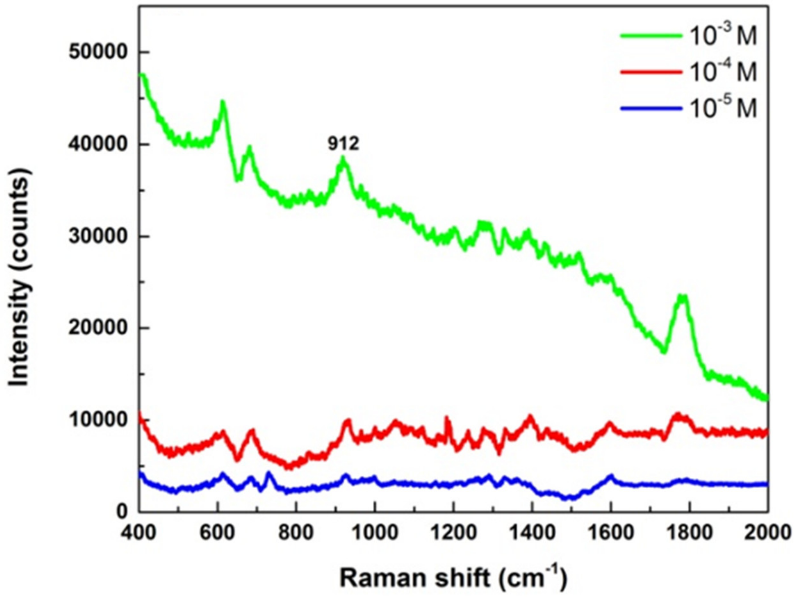

Figure S5. SERS spectra of glucose with different concentrations on Ag-AAO substrates. The laser wavelength of 532 nm, lens of 50x, and exposure time of 5 s were used for the SERS measurement.

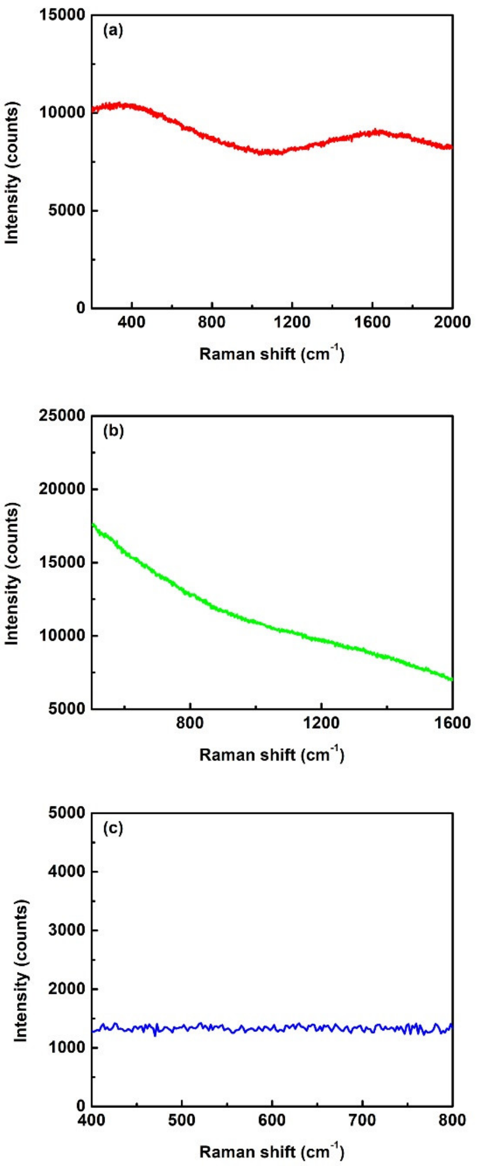

Figure S6. Raman spectra of 10^{-3} M (**a**) R6G, (**b**) eosin Y and (**c**) adenine without SERS substrates. No Raman peak is observed for the all three spectra. The spectra are pristine with neither intensity shift nor background subtraction. The laser wavelength of 532 nm, lenses of 10x for (**a**) and 50x for (**b**) and (**c**), and exposure times of 3 s for (**a**) and (**c**) and 1 s for (**b**) were used for the measurement.

Table S1. Recent development on AAO-based SERS substrates.

SERS Substrate	Sensed molecule	AAO fabrication process /growing metal	Excitation wavelength	Detection limit	Reference [1]/year
Ag-AAO substrate	Rhodamine 6G<br>Eosin Y<br>Adenine<br>Methylene blue	One-step anodization/Ag	532 nm	10^{-11} M<br>10^{-9} M<br>10^{-7} M<br>10^{-7} M	This work /2022
Au nanoparticles layer on porous AAO	Rhodamine 6G	Two-step anodization/Au	633 nm	10^{-11} M	[41]/2021
Au/PDMS/AAO SERS substrate	Patulin	Two-step anodization/Au	785 nm	8.5×10^{-11} M	[42]/2020
Canonical AAO templated Au-NPs array	Myoglobin	Two-step anodization/Au	785 nm	0.01 ppm	[43]/2019
Pt-MF-AAO SERS substrate	Methylene blue	One-step anodization/Pt	532 nm	10^{-9} M	[44]/2022
Waffle-like AAO supporting the well-dispersed Ag nanoparticles	Chloramphenicol	Two-step anodization/Ag	633 nm	4.0×10^{-9} M	[45]/2020
AuNSs@Ag@AAO substrates	0.4 μm polystyrene	Two-step anodization/Ag and Au	633 nm	50 ppm	[46]/2021
PDMS@AAO complex substrate	Aflatoxin B1<br>Deoxynivalenol<br>Zearalenone	Two-step anodization/Au	785 nm	1.8 ng/mL<br>47.7 ng/mL<br>24.8 ng/mL	[47]/2019
Multi-layer nanoarrays sandwiched by AAO membranes	4-aminothiophenol	Commercial AAO/Ag	514 nm	10^{-10} M	[48]/2018
Silver-nanoparticle arrays based on AAO membranes	Rhodamine 6G<br>Tetracycline<br>Dicyandiamide	Two-step anodization/Ag	785 nm	10^{-10} M<br>10^{-9} M<br>10^{-7} M	[49]/2020
Gold nanobipyramids	Dopamine	Commercial AAO/Au	785 nm	6.5×10^{-9} M	[50]/2019
Gold nanobipyramids	Aflatoxin B1<br>Rhodamine 6G	Commercial AAO/Au	785 nm	1.5 mg/L<br>10^{-6} M	[51]/2020

References

41. Cigarroa-Mayorga, O.E.; Gallardo-Hernandez, S.; Talamas-Rohana, P. Tunable Raman scattering enhancement due to self-assembled au nanoparticles layer on porous aao: The influence of the alumina support. Appl. Surf. Sci. **2021**, 536, 147674.
42. Zhu, Y.Y.; Wu, L.; Yan, H.; Lu, Z.C.; Yin, W.M.; Han, H.Y. Enzyme induced molecularly imprinted polymer on SERS substrate for ultrasensitive detection of patulin. *Anal. Chim. Acta* **2020**, *1101*, 111-119.
43. Muhammad, M.; Shao, C.S.; Huang, Q. Label-free SERS diagnostics of radiation-induced injury via detecting the biomarker Raman signal in the serum and urine bio-samples based on Au-NPs array substrates. *Spectrochim. Acta A Mol. Biomol. Spectrosc.* **2019**, *223*, 117282.
44. Yu, C.Y.; Chung, C.K. Novel irregular pore peripheral plasmonic mechanism of nanocomposite metal-nanoporous AAO using new facile one-step anodization and pore widening for high SERS enhancement. *Appl. Surf. Sci.* **2022**, *580*, 152252.
45. Xiao, D.F.; Jie, Z.S.; Ma, Z.Y.; Ying, Y.; Guo, X.Y.; Wen, Y.; Yang, H.F. Fabrication of homogeneous waffle-like silver composite substrate for Raman determination of trace chloramphenicol. *Mikrochim Acta* **2020**, *187*, 593.
46. Le, Q.T.; Ly, N.H.; Kim, M.K.; Lim, S.H.; Son, S.J.; Zoh, K.D.; Joo, S.W. Nanostructured Raman substrates for the sensitive detection of submicrometer-sized plastic pollutants in water. *J. Hazard. Mater.* **2021**, *402*, 123499.
47. Li, J.J.; Yan, H.; Tan, X.C.; Lu, Z.C.; Han, H.Y. Cauliflower-inspired 3d SERS substrate for multiple mycotoxins detection. *Anal. Chem.* **2019**, *91*, 3885-3892.
48. Zhao, C.C.; Zhu, Y.; Chen, L.; Zhou, S.X.; Su, Y.Q.; Ji, X.; Chen, A.Q.; Gui, X.C.; Tang, Z.K.; Liu, Z.W. Multi-layer nanoarrays sandwiched by anodized aluminium oxide membranes: an approach to an inexpensive, reproducible, highly sensitive SERS substrate. *Nanoscale* **2018**, *10*, 16278-16283.
49. Muhammad, M.; Yan, B.; Yao, G.H.; Chao, K.L.; Zhu, C.H.; Huang, Q. Surface-enhanced Raman spectroscopy for trace detection of tetracycline and dicyandiamide in milk using transparent substrate of Ag nanoparticle arrays. *ACS Appl. Nano Mater.* **2020**, *3*, 7066-7075.

50. Lin, B.Y.; Chen, J.M.; Kannan, P.; Zeng, Y.B.; Qiu, B.; Guo, L.H.; Lin, Z.Y. Rapid synthesis of a highly active and uniform 3-dimensional SERS substrate for on-spot sensing of dopamine. *Mikrochim. Acta* **2019**, *186*, 260.
51. Lin, B.Y.; Kannan, P.; Qiu, B.; Lin, Z.Y.; Guo, L.H. On-spot surface enhanced Raman scattering detection of Aflatoxin B-1 in peanut extracts using gold nanobipyramids evenly trapped into the AAO nanoholes. *Food Chem.* **2020**, *307*, 125528.
